# Cell-Surface PCNA Is Co-Expressed with Biomarkers of Stemness and Immunosuppression in Glioblastoma

**DOI:** 10.3390/cancers17243903

**Published:** 2025-12-06

**Authors:** Luke C. Cooksey, Tamara Hoteit, Ezek Mathew, Nirupama A. Sabnis, Rob D. Dickerman, Pankaj Chaudhary, Porunelloor A. Mathew

**Affiliations:** 1 Texas College of Osteopathic Medicine, UNT Health, Fort Worth, TX 76107, USA; lukecooksey@my.unthsc.edu (L.C.C.); ezekmathew@my.unthsc.edu (E.M.); rob.dickerman@unthsc.edu (R.D.D.); 2 Department of Microbiology, Immunology and Genetics, College of Biomedical and Translational Sciences, UNT Health, Fort Worth, TX 76107, USA; tamarahoteit@my.unthsc.edu (T.H.); nirupama.sabnis@unthsc.edu (N.A.S.); pankaj.chaudhary@unthsc.edu (P.C.)

**Keywords:** glioblastoma, biomarker, PCNA, stemness, immunosuppression, immunotherapy, natural killer cells

## Abstract

**Background/Objectives**: Glioblastoma (GBM) is a lethal form of primary brain tumor. There has been minimal improvement in overall GBM survival in recent years. To increase survival in patients with GBM, it is important to study novel GBM molecular antigens to form the basis of better diagnostics, prognostic measures, and therapeutic advancements. Our goal is to find more robust GBM-specific antigenic biomarkers to eventually improve GBM outcomes. Here, we initiated an investigation into cell-surface PCNA (csPCNA), a potential GBM biomarker and antigenic target. **Methods**: We utilized flow cytometry, imaging flow cytometry, and cell-surface Western blot to identify the expression of csPCNA on GBM cell lines (LN-229, LN-18) and a primary patient-derived tumor specimen. We then employed flow cytometry to study the associative co-expression of csPCNA with other biomarkers of GBM stem cells (CD44, CD49f) and GBM immunosuppression (PD-L1, TGFβRII). **Results**: We elucidated that LN-229, LN-18, and the primary GBM patient cells express csPCNA. We found that csPCNA is co-expressed with CD44, CD49f, PD-L1, and TGFβRII on the primary patient-derived GBM specimen. **Conclusions**: Our findings that csPCNA is expressed in GBM and is co-expressed with stem cell and immunosuppressive biomarkers indicate that csPCNA may be a potentially useful clinical–pathological biomarker for GBM stemness and immunosuppression.

## Simple Summary

Glioblastoma (GBM) is a deadly form of brain cancer that has seen little improvement in overall survival in the past few decades. To improve overall patient survival for those with GBM, it is paramount to develop a better understanding of how GBM grows, resists treatment, and interacts with the immune system. This improved understanding lays the groundwork for better diagnostic tools, prognostic predictors, and treatment options. With the eventual goal of finding better GBM-specific protein biomarkers to aid in understanding and treating GBM, we initiated this investigation into cell-surface proliferating cell nuclear antigen (csPCNA), a potential GBM biomarker. We found that csPCNA is expressed on commercially available GBM cell lines and in a primary patient sample. We also found that csPCNA was associated with other protein biomarkers of GBM stem cells and GBM immune suppression. This early investigation lays the groundwork for future studies of csPCNA as a potential clinical–pathological GBM biomarker and as a promising treatment target.

## 1. Introduction

Malignant gliomas are the most common primary brain tumor in adults worldwide. The most common subtype of glioma in adults is termed ‘glioblastoma’ (GBM), named by the most recent World Health Organization (WHO) guidelines as Isocitrate Dehydrogenase (IDH)-wild-type glioblastoma [1]. Most often, tumors grow to large sizes within the cerebral hemispheres and can even cross hemispheres via the corpus callosum, forming a ‘butterfly glioma’ [1]. The tumors present with varying symptoms based on the precise location of the tumor in the brain and due to the size of the tumor. Overall, GBM has a very poor prognosis with a median survival time between 12–23 months following diagnosis and a five-year survival rate between 5–10%, with a mean age of onset at roughly 65 years [1,2]. GBM tumors do not metastasize except in exceedingly rare cases [3]. Standard of treatment for GBM involves maximal safe surgical tumor resection, often followed by concurrent radiation and chemotherapy [4]. Temozolomide (TMZ) is the most common chemotherapy used to treat GBM and is given to patients deemed (via pathological analysis) to possess a methylated O-6-methylguanine-DNA methyltransferase (MGMT) promoter tumor type. Patients with unmethylated MGMT status typically do not respond as effectively to TMZ [5]. Other drugs used for GBM treatment have included procarbazine, lomustine, vincristine, and bevacizumab [1,6,7,8,9,10]. Despite best efforts, there has not been an overall improvement in long-term GBM patient survival even with the introduction of immunotherapy [11,12,13]. Contrary to reflex pessimism, there are many new and exciting immunotherapeutic avenues being explored in basic science contexts and clinical trials.

GBM tumor biology is unique in the world of cancer biology. The peculiarities of GBM biology account for many of the challenges surrounding tumor treatment. These unique problems for treatment include (but are not limited to) the blood–brain barrier (BBB), the genotypic and phenotypic heterogeneity of GBM cancers, the immune-privileged status of the brain, and the highly immunosuppressive tumor microenvironment—perhaps the single greatest barrier to immunotherapy success [10,14]. GBM has a multitude of distinct biomarkers that give insight into an individual GBM tumor’s biology, many of which are relevant to prognosis, drug and treatment responses, invasiveness, immunosuppressive capacity, and more [15,16,17]. In our current study, we are restricting the discussion to several biomarkers of stem cell capacity (CD44, CD49f) and immunosuppression (Transforming Growth Factor-beta receptor II, TGFβRII, and Programmed Cell Death Ligand-1, PD-L1). CD44 is a well-characterized transmembrane glycoprotein that is used as a common biomarker for cancer stem cells in many cancers. In GBM, high CD44 expression has previously been shown to be a biomarker for glioma stem cells and indicates cells that are highly invasive [18,19,20]. CD49f (integrin ⍺6) is a laminin receptor that has also been shown to be a reliable biomarker for glioblastoma stem cells. CD49f expression has been demonstrated to not only correlate with the stem cell capacities of GBM but also to predict therapeutic resistance and promote tumor invasion [21,22,23]. PD-L1 is a well-established biomarker for immune evasion and poor prognosis in many cancers, including GBM. PD-L1 serves as the cancer-expressed component of the PD-1/PD-L1 immune checkpoint, which inhibits cell-mediated destruction of cancer cells. In GBM, PD-L1 expression has been demonstrated to correlate with immune evasion via immune suppression and tumor aggression (by allowing invasive GBM cells to survive immune surveillance). High PD-L1 expression in GBM is associated with a poorer prognosis and lower overall survival [24,25,26,27,28]. TGFβRII is expressed in glioblastoma at high levels in some subsets of tumor cells, especially in glioma stem cells. TGFβRII expression in glioblastoma has been shown to strongly correlate with immunosuppression and an overall immunosuppressive microenvironment through various molecular mechanisms. It should be noted that while we are using each biomarker for specific phenotypic purposes, the expression of each biomarker does not fit neatly into a single category, and all of these biomarkers correlate with stemness, aggressiveness, and immunosuppressive capacity to varying degrees. For example, TGFβRII correlates with immunosuppression but also indicates stemness and increased tumor aggression [29,30,31].

For improvements in GBM diagnosis, prognosis, and treatment outcomes, new targets for immunotherapy and new prognostic biomarkers are being explored [15,32]. One avenue of pursuing improvements in GBM outcomes focuses on GBM’s interaction with natural killer (NK) cells [33]. NK cells are a type of innate lymphoid cell that carry out two important immune functions: controlled cytotoxicity and cytokine production. NK cells have a unique capacity of natural cytotoxicity, which enables them to destroy cells in an induced manner, without the need for a specific NK-receptor. They also produce cytokines, which activate and bolster anticancer immunity. NK cells can also be activated to kill target cells via a powerful process known as antibody-dependent cellular cytotoxicity (ADCC). In ADCC, NK cells are stimulated by target-bound antibodies. Following stimulation, the NK cell induces apoptosis of the target cell; for this reason, ADCC has been the focus of multiple immunotherapeutic attempts in cancer treatment. NK cell antitumor immunity is often regulated through a delicate balance of stimulatory and inhibitory signals sent into NK cells while interacting with the surfaces of potential target cells [34,35]. Activated NK cells can enter the central nervous system and interact with the surrounding tissue, including immune surveillance of GBM [36]. Prior studies have demonstrated CD16-postive NK cells are associated with higher overall survival in GBM [37,38]. However, the highly immunosuppressive tumor microenvironment can render NK cells inactive. Newer immunotherapeutic strategies, such as the use of chimeric antigen receptor (CAR-NK) cells, have great potential for improving patient outcomes because these cells can be engineered to be highly specific to GBM antigens and be resistant to an immunosuppressive environment [39,40]. Our group has previously studied multiple examples of these specific interactions, which drive NK activity in the context of multiple cancers. One of the previously studied interactions includes the interaction of the NKp44 receptor with cell-surface PCNA (csPCNA). csPCNA binding with the NKp44 receptor leads to an inhibitory signal being sent into the NK cell [41,42]. Because this signal inhibits NK cell anticancer activity, this signal may be a path of immune evasion for multiple cancers. Normally, PCNA is expressed as a homotrimer in the nuclei of rapidly dividing cells, where it is important in the functions of DNA polymerases and the cell cycle. Notably, when PCNA is expressed on the surfaces of cancers, it is expressed in a monomeric form, where it mediates the inhibitory signals into NK cells (via the interaction with NKp44) [41]. Our group has shown that csPCNA forms a complex with HLA class I molecules (HLA-A, HLA-B, or HLA-C) to mediate inhibitory signals into NK cells via the NKp44 receptor [43]. This effect has been shown to occur in multiple cancers [43,44,45,46,47,48]. Of note, csPCNA has also been shown to be expressed on the surface of the U251 glioma cell line [41]. In this study, we sought to explore the potential of csPCNA as a clinical–pathological biomarker for GBM by examining the associations of csPCNA co-expression with other established biomarkers of GBM stemness and immunosuppression. We began by exploring whether LN-229 and LN-18 cell lines expressed csPCNA and whether primary patient-derived GBM cells express csPCNA. We then examined co-expression of csPCNA with biomarkers that indicate pro-cancer phenotypes: cancer stem cells and immunosuppression. The biomarkers we studied in co-expression with csPCNA were CD44, CD49f, PD-L1, and TGFβRII.

## 2. Materials and Methods

### 2.1. Cell Culture

Human glioblastoma cell lines LN-229 (CRL-2611^™^) and LN-18 (CRL-2610^™^) cell lines were acquired from the American Type Culture Collection (ATCC, Manassas, VA, USA). Both LN-229 and LN-18 cultured using Dulbecco’s modified Eagle’s medium (DMEM, Gibco™, Grand Island, NY, USA) supplemented with the addition of 10% fetal bovine serum (FBS; R&D Systems, S11150), 1% sodium pyruvate (Gibco™, Grand Island, NY, USA), and 1% penicillin, streptomycin, and Amphotericin B (Antibiotic-Antimycotic, Gibco™, Grand Island, NY, USA). Fresh, primary patient-derived glioblastoma cells (PCCLs) were provided directly from surgery by Dr. Rob Dickerman and Ezek Mathew; they were isolated by Dr. Nirupama Sabnis for cell culture and experiments. PCCLs were cultured using Gibco™ 1× DMEM with GlutaMAX™ Supplement (Catalog No. 10566016) with 4.5 g/L D-glucose, 10% FBS (R&D Systems, S11150), and 1% penicillin, streptomycin, and Amphotericin B (Antibiotic-Antimycotic, Gibco™, Grand Island, NY, USA). All the previously mentioned cells were cultured in standard T-25, T-75, and T-175 sterile culture flasks. Cells were maintained in an incubator set to 37 °C for temperature and 5% CO_2_ humidified air. Passaging of cells was performed by the addition of 1× PBS with 10 mM ethylenediaminetetraacetic acid (EDTA) solution and subsequent incubation until the cells naturally detached with gentle force.

### 2.2. Isolation of Patient-Derived Glioblastoma Cells

GBM tissue specimens were obtained during surgery under the provision of an Institutional Review Board (IRB)-approved protocol with informed patient consent. All samples were de-identified before further processing. Tumor cell dissociation and isolation were carried out via modified versions of previously described protocols [49,50]. Briefly, the sample was transferred to a sterile dish and finely minced with a razor blade. The tissue fragments were rinsed in cold phosphate-buffered saline (PBS, pH 7.4) and collected into a 15-mL conical tube by careful pipetting 3–4 times. After allowing the pieces to settle at the bottom of the tube while on ice for 1 min, the supernatant was discarded, and the pellet was resuspended in 5 mL Hank’s balanced salt solution (HBSS). The resuspended sample was then moved into a new 15-mL conical tube and digested with collagenase (100 U/mL in HBSS) for 90 min with the tube in a 37 °C water bath. Digestion was quenched by rapidly adding ice-cold fetal bovine serum (FBS; R&D Systems, S11150). The sample was then processed immediately for cell isolation and culture. Cells were then seeded into T-75 flasks containing DMEM/F12 medium supplemented with 20% FBS and 1% penicillin, streptomycin, and Amphotericin B (Antibiotic-Antimycotic, Gibco™, Grand Island, NY, USA). The flasks were then maintained in a humidified cell culture incubator at 37 °C with 5% CO_2_. After three days, when adherent cells were observed, the cell cultures were then subsequently expanded. Once 80% confluence had been achieved, cells were then passaged, and stocks were cryopreserved. Following a period of growth optimization, PCCLs were subsequently maintained in culture using Gibco™ 1× DMEM with GlutaMAX™ Supplement (Catalog No. 10566016) with 4.5 g/L D-glucose, 10% FBS, and 1% penicillin, streptomycin, and Amphotericin B.

### 2.3. Flow Cytometry

LN-229, LN-18, and PCCL cells were grown to appropriate (80–90%) confluence in sterile culture flasks before beginning the experimental protocol. Cells were harvested using the addition of 1× PBS-EDTA and subsequent incubation until detachment occurred with gentle force. Cells were mixed with Trypan Blue (Gibco™, Grand Island, NY, USA) and counted using a hemacytometer to obtain 10^6^ cells per sample group. Cells were then stained with primary antibodies with an attached fluorophore or isotype control antibodies with corresponding fluorophores in the dark at 4 °C for 30 min. All antibodies were specific for human antigens. The antibodies used during experiments were as follows: PE-anti-PCNA (5 μL per 10^6^ cells; BioLegend, Clone #PC10); PE Mouse IgG2a κ Isotype Control (BioLegend, Clone #MOPC-21, San Diego, CA, USA); FITC-anti-CD44 (5 μL per 10^6^ cells; BioLegend, Clone #BJ18); FITC Mouse IgG1 κ Isotype Control (BioLegend, Clone #MOPC-21, San Diego, CA, USA); Pacific Blue™ anti-human CD49f (5 μL per 10^6^ cells; BioLegend, Clone #GoH3); Pacific Blue™ Rat IgG2a κ Isotype Control (BioLegend, Clone #RTK2758, San Diego, CA, USA); Brilliant Violet 421™ anti-human CD274 (B7-H1, PD-L1) (5 μL per 10^6^ cells; BioLegend, Clone #MIH3); Brilliant Violet 421™ Mouse IgG1 κ Isotype Control (BioLegend, Clone #MOPC-21, San Diego, CA, USA); APC anti-human TGF-β Receptor II (5 μL per 10^6^ cells; BioLegend, Clone #W17055E); APC Rat IgG2b κ Isotype Control (BioLegend, Clone #RTK4530, San Diego, CA, USA). After staining, cells were washed with and suspended in a 1× PBS-5% BSA-1 mM EDTA solution to remove unbound antibodies. Cells were then run through the Cytek Aurora Flow Cytometer located in the UNT Health Fort Worth Flow Cytometry Core Facility. FlowJo 10.8.1 software (FlowJo, LLC, Ashland, OR, USA) was used for data analysis of the completed experiments.

### 2.4. Imaging Flow Cytometry

Preparation of LN-229 and LN-18 cells for imaging flow cytometry followed the same protocol as mentioned in the previous section until the time for antibody staining and fixation. For the first set of imaging, we simply stained the surfaces of LN-229 and LN-18 cells using the standard flow cytometry protocol followed by fixation (but not permeabilization) with the Invitrogen™ FIX & PERM™ Cell Permeabilization Kit. For this, we followed the protocol to stain for csPCNA on both cell lines. We also stained for HLA-Class I molecules for comparison. Next, we used permeabilization to examine csPCNA detection against intracellular PCNA detection. Cells were divided into two groups according to permeabilization status: one group of cells was fixed and stained; the other group was fixed, permeabilized, and then stained using fluorophore-tagged antibodies. Both the fixation and permeabilization processes were carried out using the Invitrogen™ FIX & PERM™ Cell Permeabilization Kit. The first group was fixed according to the Invitrogen™ Kit protocol and then stained with primary antibodies with an attached fluorophore or isotype control antibodies with corresponding fluorophores in the dark at 4 °C for 30 min. The second group was fixed and permeabilized according to the Invitrogen™ Kit and stained with the same antibodies as the non-permeabilized in the dark at 4 °C for 30 min. All antibodies were specific for human antigens. The antibodies used during experiments were PE-anti-PCNA (5 μL per 10^6^ cells; BioLegend, Clone #PC10); PE Mouse IgG2a κ Isotype Control (BioLegend, Clone #MOPC-21, San Diego, CA, USA); APC/Cy7-anti-HLA-A, B, C (5 μL per 10^6^ cells; BioLegend, Clone #W6/32); and APC/Cy7 Mouse IgG2a (5 μL per 10^6^ cells; BioLegend, Clone #MOPC-173). After staining, cells were washed with 1× PBS-5% BSA-1 mM EDTA solution to remove unbound antibodies and then suspended in 1% paraformaldehyde until the time of the experiment. Cells were then run through the Amnis^®^ ImageStream^®^ Mk II imaging flow cytometer located in the UNT Health Fort Worth Flow Cytometry Core Facility. Luminex IDEAS^®^ software (version 6.3, Luminex, Austin, TX, USA) was used for data analysis of the completed experiments and images.

### 2.5. Cell Surface Biotinylation and Western Blot

LN-229, LN-18, and PCCL cultures were expanded until reaching 80–90% confluence before initiating experiments. For these experiments, the protocol established by Guo et al. was followed to completion [51]. Cells were rinsed with serum-free DMEM and subjected to surface biotinylation using sulfo-NHS-biotin (0.5 mg/mL; Cayman Chemicals, Ann Arbor, MI, USA) for 30 min at 37 °C in a humidified incubator. Afterward, the cells were washed four times with PBS and lysed in RIPA buffer containing protease and phosphatase inhibitors (Millipore, Burlington, MA, USA), and lysates were collected in 1.5-mL microcentrifuge tubes. Protein concentrations were determined using the BCA assay (Pierce™ BCA Protein Assay Kits, Thermo Scientific™, Waltham, MA, USA). 400–500 μg of total protein was used for subsequent steps. Surface-biotinylated proteins were enriched by overnight incubation at 4 °C with Pierce™ High-Capacity NeutrAvidin™ Agarose (Thermo Scientific). Beads were washed repeatedly with RIPA buffer supplemented with phosphatase and protease inhibitors, and bound proteins were released by resuspension in 2× Laemmli buffer, followed by boiling for 10 min. Samples were subjected to SDS-PAGE followed by immunoblot analysis. Coomassie blue staining (InstantBlue^®^, Expedeon, Abcam, ab119211) was used as a loading control. Samples were run in duplicates on the gel. One set was used for the Western and the other was used for the Coomassie stain. Protein was transferred to the nitrocellulose blot on one set, and the other gel was placed in the Coomassie stain until the distinctive blue bands developed. The following antibodies were used: anti-human PCNA (BioLegend, Clone PC10, San Diego, CA, USA) and anti-Lamin A/C (ABclonal, A19524, Woburn, MA, USA). Protein band intensities were quantified and analyzed using ImageJ software (version 10.8.1). A graphic depicting the workflow of this method is shown in Section 3.1.

### 2.6. Statistical Methods

Statistical analysis was performed for applicable experiments with the use of Prism 8.0 (GraphPad Software, San Diego, CA, USA). For specific tests used, please see the corresponding figure legend. Results were considered significant if *p* < 0.05 for the applicable experiments.

## 3. Results

### 3.1. Glioblastoma Cell Lines Express csPCNA

**Figure 1:**
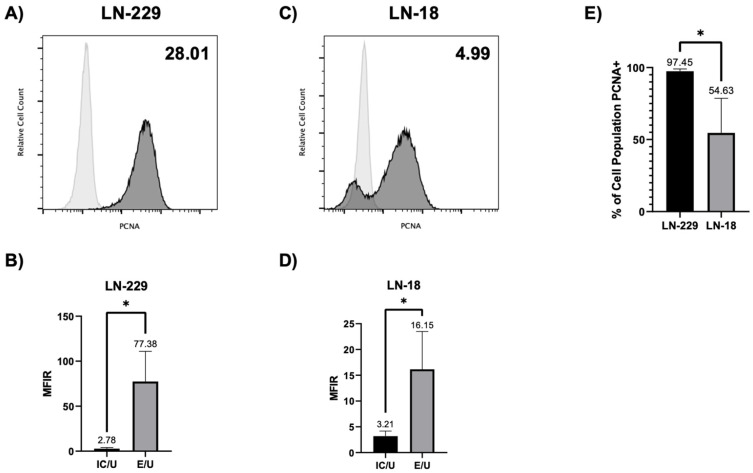
LN-229 and LN-18 cells express csPCNA. (**A**,**C**) Flow cytometry histogram displaying csPCNA detection (dark gray curve) against the PE-isotype control (light gray curve) for LN-229 (**A**) and LN-18 (**C**) cells. Mean MFIR of csPCNA/isotype control for all experiments is displayed in the top right corner of each histogram. (**B**,**D**) Bar graphs comparing overall mean MFIRs of isotype control/unstained control (IC/U) against mean MFIRs of csPCNA/unstained control (E/U) for LN-229 (**B**) and LN-18 (**D**) cells. (**E**) Bar graph displaying the comparison of the overall means of csPCNA-positive cell populations between LN-229 and LN-18 cell lines. Bar graph data are presented as mean + SD (*n* ≥ 4). Numerical values above columns and error bars represent the mean of each group. *p*-values were calculated by using unpaired two-tailed Student’s *t*-tests (**B**,**D**,**E**). * = *p* < 0.05.

**Figure 2:**
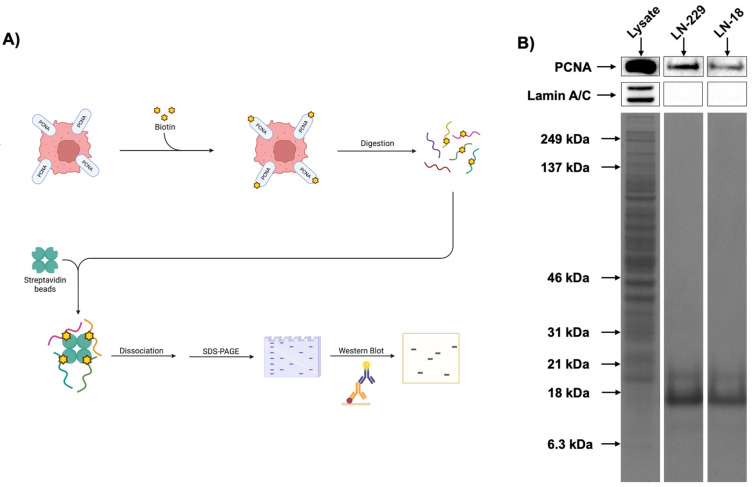
Validation of LN-229 and LN-18 csPCNA expression by cell surface protein biotinylation and Western blot. (**A**) Graphic displaying the summarized workflow of sequential cell surface biotinylation followed by SDS-PAGE and Western blot. (**B**) Western blots comparing whole cell lysate, LN-229, and LN-18. Cell lysate was not biotinylated—blot represents detection from all cell layers and was used as a positive control for PCNA and Lamin A/C. Lamin A/C detection was used as a location-specific control for nuclear PCNA; detection of Lamin A/C meant nuclear proteins (and therefore nuclear PCNA) were present. Lack of Lamin A/C detection indicated no nuclear contamination in the protein sample. Tall columns below PCNA and Lamin A/C blots represent Coomassie blue protein stains displaying total protein present in the sample. The original uncropped Western blot figures can be found in the Supplementary File S1.

To examine whether glioblastoma cells available to us (cell lines other than U251) expressed csPCNA, we first employed standard flow cytometry (Figure 1). Intact, non-permeabilized LN-229 and LN-18 cells were stained for csPCNA expression using anti-human PCNA antibodies and corresponding isotype controls. Since healthy, non-malignant cells do not express csPCNA, any positive measurement was deemed to indicate expression. Mean fluorescence intensity (MFI) was determined for each sample in each cell line as the geometric mean of curves generated by each fluorophore. MFI ratios (MFIR) were generated for each cell line to determine the proportional differences in MFI between groups stained with anti-PCNA antibodies and corresponding fluorophore-tagged isotype control antibodies. MFIRs ≤ 1 indicate no difference between the isotype control and PCNA-specific antibody, indicating csPCNA is likely not present. MFIRs > 1 indicate the csPCNA is likely present on the cell surface when comparing anti-PCNA staining with the corresponding isotype control. For LN-229, multiple experimental tests demonstrated an overall MFIR mean of 28.01 when csPCNA MFI is compared to isotype control MFI, showing the detection of PCNA on the surface of the cells (Figure 1A,B). For LN-18, multiple experimental runs displayed an overall mean MFIR of 4.99 when csPCNA MFI is compared to isotype control MFI, showing detection of csPCNA (Figure 1C,D). To further verify the presence of csPCNA in both cell lines, we performed a sequential cell surface biotinylation and Western blot followed by imaging flow cytometry for visualization of csPCNA on the cell surface. The surfaces of LN-18 and LN-229 cells were biotinylated to mark out surface proteins only (Figure 2A). The biotinylated surface proteins were then isolated and subjected to an immunoblot to assess for the presence of csPCNA. Lamin A/C was used as a negative control for the detection of nucleus-based PCNA in the biotinylated samples. A whole cell lysate was also used as a positive control for a high degree of PCNA detection from the nucleus and cytosol. csPCNA was detected on both cell lines, while Lamin A/C was negative, verifying that the detected PCNA was indeed from the cell surface and not from the nucleus (Figure 2B).

**Figure 3:**
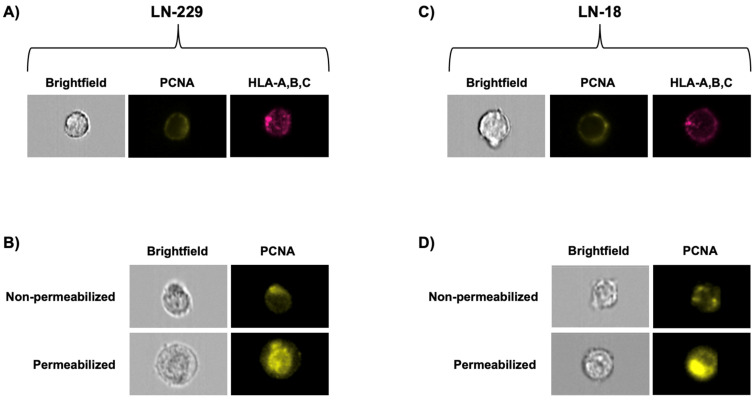
Imaging flow cytometry panels displaying csPCNA on LN-229 (**A**,**B**) and LN-18 (**C**,**D**) cells. (**A**,**C**) Images showing Brightfield (greyscale), PE-anti-PCNA (yellow), and APC/Cy7-anti-HLA-A, B, C (pink) detection on LN-229 (**A**) and LN-18 (**C**) samples. (**B**,**D**) Comparison of PCNA (yellow) detection on images obtained in non-permeabilized vs. permeabilized LN-229 (**B**) and LN-18 (**D**) cells. Permeabilization was used to detect nuclear and cytosolic PCNA for comparison. Images were obtained using an Amnis^®^ ImageStream^®^ Mk II imaging flow cytometer.

To obtain visual data of csPCNA on LN-229 and LN-18 cells, we performed imaging flow cytometry (Figure 3). We first performed imaging cytometry to verify csPCNA expression in fixed, non-permeabilized LN-229 and LN-18 cells (Figure 3A,C). We detected csPCNA in both cell lines and used HLA-A, HLA-B, and HLA-C as a visual qualitative comparison, as csPCNA forms a surface complex with HLA class I molecules. To compare surface PCNA expression with intracellular expression of PCNA, we permeabilized one group of LN-229 and one group of LN-18, followed by PCNA staining. The obtained images of non-permeabilized cells demonstrated a detectable amount of csPCNA specific to the cell surface with no intranuclear detection (Figure 3B,D). In both cell lines with permeabilized membranes, a much higher intensity of PCNA detection was consistently found intracellularly when compared to the surface staining, as expected.

### 3.2. Glioblastoma Patient-Derived Cells Express csPCNA

**Figure 4:**
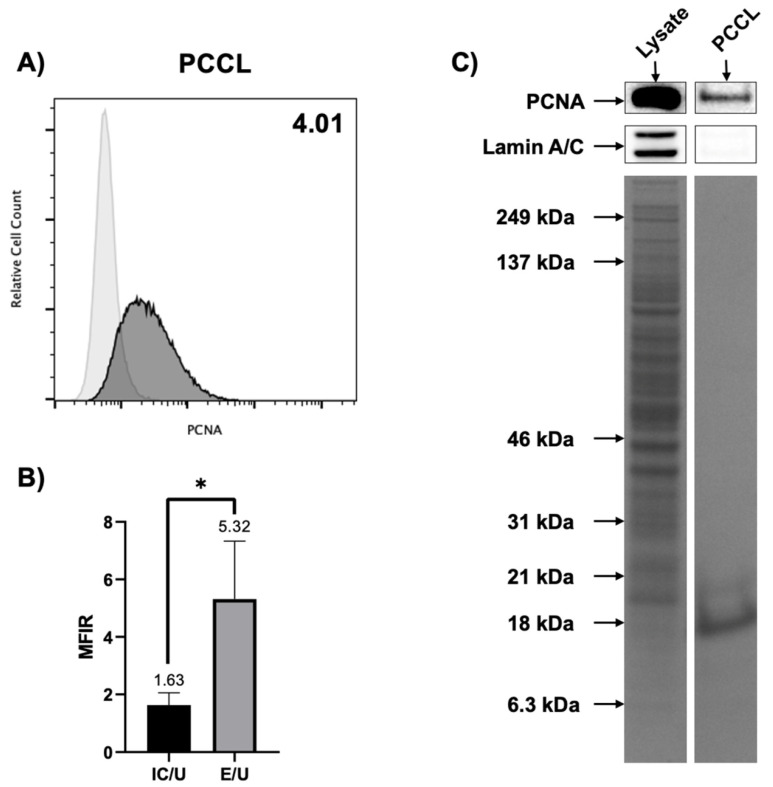
Patient-derived glioblastoma cells (PCCLs) express csPCNA. (**A**) Flow cytometry histogram displaying csPCNA detection (dark gray curve) against the PE-isotype control (light gray curve) for PCCL cells. Mean MFIR of csPCNA/isotype control for all experiments is displayed in the top right corner of the PCCL histogram. (**B**) Bar graph comparing the overall mean + SD of isotype control/unstained control (IC/U) MFIR against csPCNA/unstained control (E/U) MFIR for PCCL cells. Numerical values above columns and error bars represent the mean of each group. (**C**) Western blots comparing PCNA detection in whole cell lysate (**left**) with PCCLs (**right**). The same protocol and control method used for Figure 2 was used for this experiment. Lamin A/C was again used as a control for nuclear protein contamination. Tall columns below PCNA and Lamin A/C blots display the Coomassie blue total sample protein stains. Bar graph data (**B**) are presented as mean + SD (*n* ≥ 4). *p*-value was calculated by using unpaired two-tailed Student’s *t*-tests (**B**). * = *p* < 0.05. The original uncropped Western blot figures can be found in the Supplementary File S1.

After verifying the expression of csPCNA on LN-229 and LN-18 cells, we tested whether these results translated to primary tumor samples freshly obtained from a patient. GBM tumor samples were obtained during tumor resection surgery. GBM cells were isolated to obtain viable patient-derived cells (PCCLs) following the protocol outlined in the methods section. When PCCLs were established in cell culture, we subjected the cells to the same experiments previously performed with the LN-229 and LN-18 cell lines. Flow cytometry was first used to test for the detection of csPCNA. Multiple experiments demonstrated an overall MFIR mean of 4.01 (comparing csPCNA detection to isotype control detection), confidently indicating the presence of csPCNA on the cell surface in a patient sample (Figure 4A,B). We then performed cell surface biotinylation and Western blot to further isolate cell surface proteins and verify the presence of csPCNA. csPCNA on PCCLs was verified and was found comparable to that of LN-18 cells, all with negative Lamin A/C detection (Figure 4C).

### 3.3. csPCNA Is Co-Expressed with Biomarkers of Stemness and Immunosuppression

**Figure 5:**
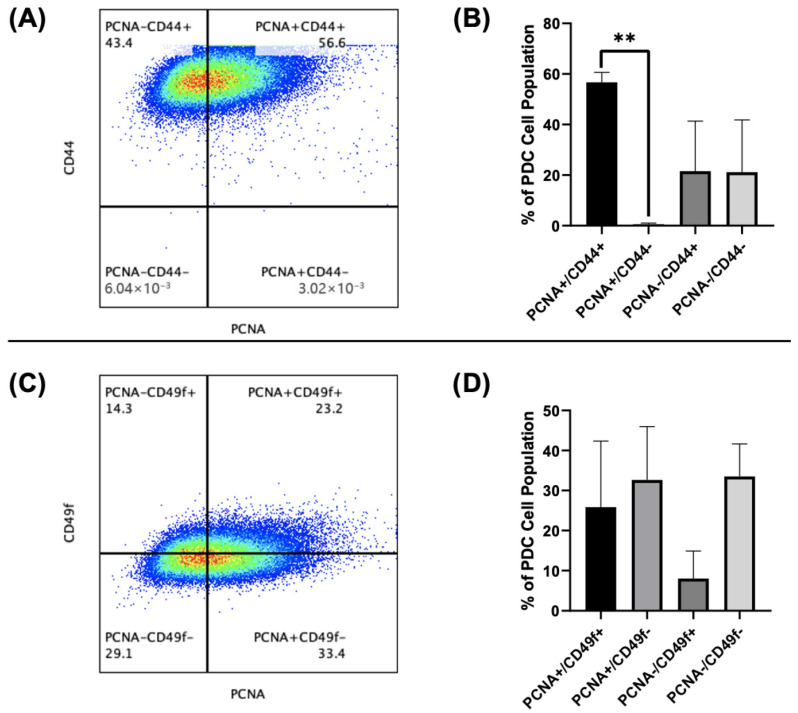
csPCNA co-expression with glioma stem cell biomarkers CD44 (**A**,**B**) and CD49f (**C**,**D**) in primary PCCLs. (**A**) Flow cytometry gated plot displaying cells that are single-positive CD44 (PCNA-CD44+), double-positive for PCNA and CD44 (PCNA+CD44+), single-positive PCNA (PCNA+CD44-), and double-negative (PCNA-CD44-). (**B**) Bar graph displaying percentage mean + SD for each PCNA or CD44 gated population as a percentage of total PCCLs. (**C**) Flow cytometry gated plot displaying cells that are single-positive CD49f (PCNA-CD49f+), double-positive for PCNA and CD49f (PCNA+CD49f+), single-positive PCNA (PCNA+CD49f-), and double-negative (PCNA-CD49f-). (**D**) Bar graph displaying percentage mean + SD for each PCNA or CD49f gated population as a percentage of total PCCLs. Numbers displayed under each quadrant label indicate the percentage of total cells meeting the parameters of that quadrant. *p*-value was calculated using a one-way ANOVA with a Tukey multiple comparisons test (**B**). = *p* < 0.05; ** = *p* < 0.01.

To explore the initial question of csPCNA as a potential biomarker, we examined the co-expression of csPCNA in comparison with other previously established biomarkers of glioma stem cells and immunosuppression in PCCLs via flow cytometry. For stem cell phenotype testing, we examined whether csPCNA was co-expressed with CD44 and CD49f (integrin ⍺6) (Figure 5A–D). We found that csPCNA was co-expressed with CD44 and CD49f on PCCLs, though there was a stronger csPCNA co-expression relationship with CD44 (Figure 5A,B). In examining csPCNA and CD44 expression in PCCLs, a mean of 56.73% (range: 52.9–60.7%) of PCCLs were double-positive for csPCNA and CD44 expression, with a mean of 0.55% (range: 0.003–0.93%) being single-positive csPCNA cells and a mean of 21.55% (range: 4.8–43.4%) single-positive CD44 cells (Figure 5A,B). In testing specifically for csPCNA and CD49f co-expression in PCCLs, a mean of 25.83% (range: 10.8–43.5%) of cells were double-positive for csPCNA and CD49f expression (Figure 5C,D). A mean of 32.63% (range: 18.9–45.6%) of PCCLs were single-positive for csPCNA, and a mean of 8.00% (range: 0.63–14.3%) were positive for CD49f only.

**Figure 6:**
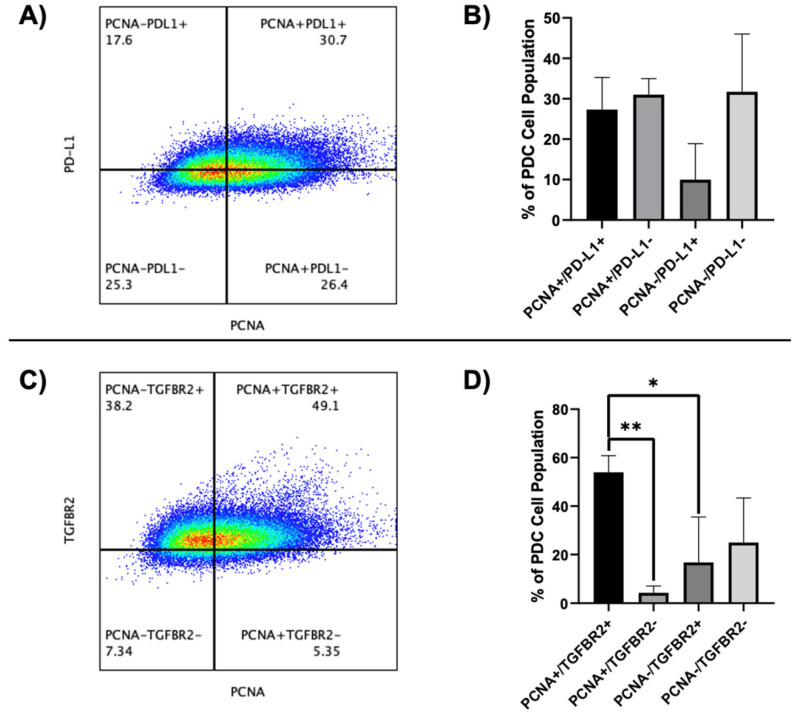
csPCNA co-expression with immunosuppression biomarkers PD-L1 (**A**,**B**) and TGFBR2 (**C**,**D**) in primary PCCLs. (**A**) Flow cytometry gated plot displaying cells that are single-positive PD-L1 (PCNA-PDL1+), double-positive for PCNA and PD-L1 (PCNA+PDL1+), single-positive PCNA (PCNA+PDL1-), and double-negative (PCNA-PDL1-). (**B**) Bar graph displaying percentage mean + SD for each PCNA or PD-L1 gated population as a percentage of total PCCLs. (**C**) Flow cytometry gated plot displaying cells that are single-positive TGFβRII (PCNA-TGFβRII+), double-positive for PCNA and TGFβRII (PCNA+TGFβRII+), single-positive PCNA (PCNA+TGFβRII-), and double-negative (PCNA-TGFβRII-). (**D**) Bar graph displaying percentage mean + SD for each PCNA or TGFBR2 gated population as a percentage of total PCCLs. Numbers displayed under each quadrant label indicate the percentage of total cells meeting the parameters of that quadrant. *p*-value was calculated using a one-way ANOVA with a Tukey multiple comparisons test (**B**). * = *p* < 0.05; ** = *p* < 0.01.

To investigate the basic potential of csPCNA as a biomarker of immunosuppression, we investigated csPCNA co-expression in conjunction with PD-L1 (Figure 6A,B) and TGFβRII (Figure 6C,D). We observed that csPCNA was positively co-expressed with both biomarkers for immunosuppression, though csPCNA was more positively co-expressed with TGFβRII than with PD-L1. A mean of 27.30% (range: 18.2–33.0%) of PCCLs were noted to be double-positive for csPCNA and PD-L1, while a mean of 31.00% (range: 26.4–33.5%) were single-positive for csPCNA and a mean of 10.00% (range: 0.19–17.6%) single-positive for PD-L1 (Figure 6A,B). A mean of 54.00% (range: 49.1–61.8%) of PCCLs were detected to be double-positive for csPCNA and TGFβRII, while a mean of 4.31% (range: 1.14–6.45%) were single-positive for csPCNA and a mean of 16.79% (range: 3.75–38.2%) were single-positive for TGFβRII (Figure 6C,D).

## 4. Discussion

Glioblastoma (GBM) is a uniquely devastating cancer with a consistently poor prognosis [1,2]. Current efforts to improve outcomes include developing better diagnostics, identifying novel prognostic biomarkers, and engineering new treatment options. There is increasing interest in bringing the immunotherapy revolution to GBM care, with numerous clinical trials and drug discovery efforts underway [32]. In parallel with GBM immunotherapy research, there is growing momentum toward identifying new biomarkers that may improve patient outcomes by enhancing survival and reducing treatment-related toxicity [15].

GBM is well known to be an immunosuppressive malignancy [52]. Its ability to suppress the anticancer activity of natural killer (NK) cells is well documented and contributes to the tumor’s aggressive behavior [10,33,53]. Although this presents a challenge, it also creates opportunities for therapeutic innovation when the mechanisms of immunosuppression are clearly understood and leveraged to design strategies that overcome these barriers [33]. Advancing NK cell-based therapies against GBM requires deeper insight into both GBM biology and NK cell regulation. NK cell activity depends on a delicate balance of stimulatory and inhibitory signals originating from the target cell and its surrounding microenvironment [34,35].

Our laboratory has previously examined several ligand–receptor interactions that influence NK cell activity across multiple cancers. When tumors exploit inhibitory NK cell pathways, tumor-expressed inhibitory ligands may become attractive therapeutic targets. This concept has been a longstanding focus of our group. For example, CS1 (CD2 subset 1; also known as CD319, CRACC, or SLAMF7) forms a homodimer on both multiple myeloma cells and NK cells and is highly overexpressed in multiple myeloma [54,55,56,57,58,59,60]. This discovery contributed to the development of the monoclonal antibody elotuzumab (EMPLICITI^®^), now approved for treating multiple myeloma [61,62]. Similarly, LLT1 expression on cancer cells has been shown to inhibit NK cell function through interaction with NKR-P1A (CD161) across multiple malignancies [44,63,64,65,66]. More recently, csPCNA was found to form a complex with HLA-A, HLA-B, and HLA-C molecules to deliver inhibitory signals via NKp44 on NK cells [41,42,43]. Notably, csPCNA has been identified in a variety of malignant cancers but not in healthy tissues [44,45,46,47].

In this study, we initiated foundational work investigating csPCNA as a potential GBM biomarker. Our first objective was to determine whether GBM cell lines express csPCNA. Using the GBM cell lines LN-229 and LN-18, we confirmed csPCNA expression through multiple experimental approaches. Because long-established cell lines often accumulate mutations and characteristics that diverge from patient tumors [67], we next examined csPCNA expression in patient-derived cancer cells (PCCLs). Using fresh primary PCCLs, we again observed clear expression of csPCNA, supporting further investigation of its relevance in GBM.

We then assessed whether csPCNA is co-expressed with established biomarkers associated with stemness and immunosuppression [18,19,20,22,23,24,25,26,27,28,29,31]. We found that csPCNA co-localized with the glioma stem cell markers CD44 and CD49f, providing preliminary evidence that csPCNA may be associated with a stem cell phenotype in GBM. In addition, csPCNA was frequently co-expressed with PD-L1 and TGFβRII in PCCLs, suggesting a potential link between csPCNA expression and immunosuppressive signaling pathways that may contribute to adverse clinical outcomes. Nonetheless, further studies are needed to determine the functional significance of csPCNA and to assess whether it represents a clinically meaningful biomarker for GBM stemness or immunosuppression.

Overall, these data support the hypothesis that csPCNA is a potentially relevant biomarker for identifying tumor stemness and immunosuppressive phenotypes in GBM. We are not proposing csPCNA as a replacement for or equivalent to established biomarkers such as CD44, CD49f, PD-L1, or TGFβR2. Instead, the data presented here represent preliminary work demonstrating that csPCNA shares phenotypic characteristics with these well-known markers. It is reasonable to speculate that csPCNA may participate in immune evasion and may ultimately be associated with poorer patient survival. Future studies should aim to elucidate its precise functional role, evaluate its prognostic value, and investigate its potential as a therapeutic target.

## 5. Conclusions

In summary, our findings support continued investigation of csPCNA as a potential biomarker of stemness and immunosuppression in GBM. These results provide a strong rationale for initiating larger-scale studies incorporating a broader cohort of patient-derived primary samples. However, several limitations must be acknowledged. First, this study focuses on descriptive characterization rather than functional analyses. Second, the limited number of patient-derived samples restricts generalizability and statistical power.

To address these limitations, our ongoing work will include (1) detailed functional and mechanistic studies of csPCNA in GBM; (2) expansion of our patient-derived sample cohort; (3) investigation of csPCNA’s role in therapeutic resistance at the cellular level; and (4) integration of csPCNA expression data with clinical measures such as treatment response and overall survival. The findings presented here, along with future studies, will be essential for determining the utility of csPCNA as a clinical–pathological biomarker and its potential as a target for therapeutic development.

## Data Availability

The data presented in this study are available on request from the corresponding author due to privacy concerns and restrictions. The raw data supporting the conclusions of this article can be made available by the authors on reasonable request.

## Supplementary Materials

The following supporting information can be downloaded at: https://www.mdpi.com/article/10.3390/cancers17243903/s1, File S1: The original uncropped Western blot figures.

## Abbreviations

The following abbreviations are used in this manuscript:

GBM	Glioblastoma
PCCL	Patient-Derived cell
NK	Natural Killer cell
csPCNA	Cell-Surface Proliferating Cell Nuclear Antigen
HLA	Human Leukocyte Antigen
LLT1	Lectin-like Transcript 1
CD	Cluster of Differentiation
WHO	World Health Organization
IDH	Isocitrate Dehydrogenase
TGFβRII	Transforming Growth Factor-Beta Receptor 2
PD-L1	Programmed Cell Death Protein Ligand 1
ADCC	Antibody-Dependent Cellular Cytotoxicity
