# Supplementary material for: Cell-Surface PCNA Is Co-Expressed with Biomarkers of Stemness and Immunosuppression in Glioblastoma
Source: Cancers (Basel). 2025 Dec 6;17(24):3903. doi: 10.3390/cancers17243903 (PMC12730262; doi:10.3390/cancers17243903)

Supplementary Figure S1

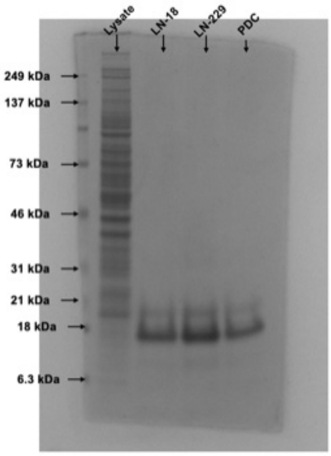

Supplementary Figure S2

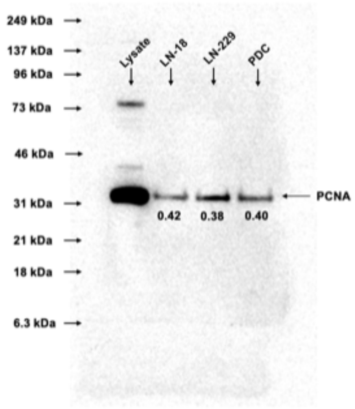

Supplementary Figure S3

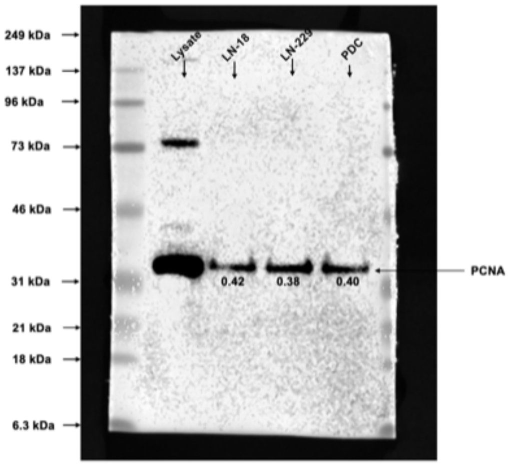

Supplementary Figure S4

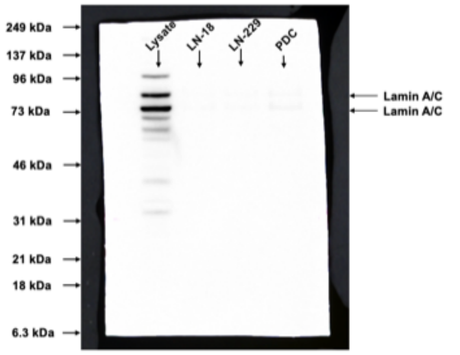

Supplementary Figure S5

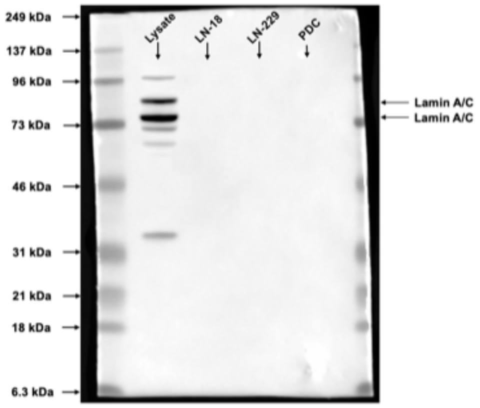

Supplement: Supplementary file 1 [file cancers-17-03903-s001.zip › cancers-4002461-supplementary.pdf]
